# The Impact of Neutrophil-to-High-Density Lipoprotein Ratio and Serum 25-Hydroxyvitamin D on Ischemic Heart Disease

**DOI:** 10.3390/jcm13216597

**Published:** 2024-11-02

**Authors:** Ewelina A. Dziedzic, Jakub S. Gąsior, Kamila Koseska, Michał Karol, Ewa Czestkowska, Kamila Pawlińska, Wacław Kochman

**Affiliations:** 1 Cardiovascular Clinic, Centre of Postgraduate Medical Education, 01-813 Warsaw, Poland; 2 Department of Pediatric Cardiology and General Pediatrics, Medical University of Warsaw, 02-091 Warsaw, Poland; 3 Faculty of Medicine, Collegium Medicum, Cardinal Stefan Wyszynski University of Warsaw, 01-938 Warsaw, Poland; 4 Department of Cardiology, Bielanski Hospital, 01-809 Warsaw, Poland

**Keywords:** NHR, HDL, vitamin D, 25-hydroxyvitamin D, ischemic heart disease

## Abstract

**Background:** This study describes the complex association between the neutrophil-to-high-density lipoprotein cholesterol ratio (NHR), 25-hydroxyvitamin D (25(OH)D) levels, and cardiovascular disease (CVD), such as stable ischemic heart disease (IHD), ST elevation myocardial infarction (STEMI), non–ST-segment elevation myocardial infarction (NSTEMI), and unstable angina (UA). **Methods:** The serum 25(OH)D concentration and NHR values were analyzed in groups of patients with chronic coronary syndrome (CCS) and acute coronary syndrome (ACS). The severity of coronary artery atherosclerosis was determined using the Coronary Artery Surgery Study (CASS) scale. **Results:** Significant differences in 25(OH)D and NHR concentrations were observed between CCS and (ACS)/STEMI patients (*p* < 0.01). Higher 25(OH)D concentrations were associated with the diagnosis of CCS, and higher NHR values with the diagnosis of ACS/STEMI. The NHR threshold for ACS was set at 0.10 (*p* < 0.001). Patients without significant coronary artery stenosis showed significantly higher 25(OH)D levels and lower NHR values (*p* < 0.01). **Conclusions:** The significant correlation between 25(OH)D, HDL, and the NHR suggests that vitamin D, through its influence on inflammatory processes and lipid metabolism, may play a role in the pathogenesis of chronic and acute coronary syndromes. The suggested bidirectional relationship between the NHR and 25(OH)D and the role of the NHR as a predictor of vitamin D levels require further well-designed studies.

## 1. Introduction

Changes in lifestyle including unhealthy eating habits and low physical activity have led to a significant increase in the incidence of cardiovascular disease (CVD). Coronary artery atherosclerosis underlying chronic coronary syndrome (CCS) is one of the most prevalent diseases, not only in developed countries but in recent years also in developing regions [1]. Data from the World Health Organization (WHO) point to CCS as the most common cause of death, causing nearly 9 million deaths worldwide in 2019 [2]. The scale of the problem indicates the need for an in-depth understanding of the genesis of the disease, and patients at risk of CVD are a priority for modern preventive cardiology.

Previous studies have well-documented the inflammatory etiology of atherosclerosis [3] and demonstrated the positive effect of immunosuppressive treatment on its course [4], highlighting the surprising effect of active smoking in young patients with STEMI (‘smoker’s paradox’) [5] and the need for further research. Identified CCS risk factors, which include dyslipidemia, are responsible for more than 90% of the likelihood of heart attack [6]. As atherosclerotic lesions are characterized by the accumulation of fat molecules in the walls of large- and medium-sized arteries, molecular studies of lipids play an important role in understanding the basis of the disease. Recent analysis of protein structure and function has led to the identification of apolipoproteins (APOs) [7]. It has been shown that APOs as non-traditional lipid markers in addition to the classical lipid fractions may represent an independent cardiovascular risk factor and a bridge in the mechanism of action of vitamin D and lipids, the most abundant subtype of white blood cells involved in each step of the atherosclerosis process [8,9,10]. Neutrophils inducing apoptosis of smooth muscle cells potentiate vascular wall inflammation and oxidative stress [11]. The number of these cells proved to be an independent predictor of the diagnosis and severity of coronary atherosclerosis [8,9], with a correlation strength equal to that of total cholesterol [8]. There was a positive association between their number and the susceptibility of the atherosclerotic plaque to rupture leading to myocardial infarction. In turn, increased numbers of these cells interacting with the endothelium and thrombocytes may lead to an increase in blood thrombotic potential and microvascular damage.

NHR—the ratio of total neutrophils to serum high-density lipoprotein (HDL) cholesterol concentration—is a new indicator for depicting the body’s level of inflammation and oxidative stress as a tool for assessing cardiovascular risk. This study reported a positive association between the NHR values and the presence of non-linear atherosclerosis with a saturation effect for an NHR value of 3.32. The results of several other papers indicate an association of the biomarker with the progression of coronary atherosclerosis [12,13,14,15] and its predictive value for the diagnosis of metabolic syndrome [16] or major adverse cardiac events (MACE) [17]. In contrast, in patients with acute coronary syndrome after treatment of total coronary artery occlusion, an elevated NHR was associated with increased cardiovascular mortality in long-term follow-up [18].

The identification of patients with an increased inflammatory risk expressed by C-reactive protein (hsCRP) levels despite a reduction in LDL cholesterol, a fraction of key importance in the pathogenesis of atherosclerosis [19], points to the urgent need to identify new factors involved in inflammatory responses of the vascular wall. The role of calcitriol in this context and, in particular, the importance of vitamin D correction in the prevention of CCS is still under debate. Vitamin D is a steroid hormone with multidirectional effects in various systems and organs [20]. The presence of the receptor for vitamin D (VDR) in the cardiovascular system in endothelial cells, vascular smooth muscle, the immune system, and cardiomyocytes [21] is responsible for the anti-inflammatory and anti-atherosclerotic properties of calcitriol [22]. It has been shown that apolipoprotein A1-mediated vitamin D increases the concentration of the HDL fraction [23]. In turn, affecting neutrophils reduces the adhesion and aggregation of these cells. Calcitriol deficiency results in impaired migration of neutrophil granulocytes, decreased synthesis of leukotriene B4, increased production of reactive oxygen species (ROS), and pro-inflammatory cytokines [24]. Vitamin D deficiency can cause damage to endothelial cells at an early stage of atherosclerosis, without clinically overt cardiovascular disease [25], and its beneficial effects on vascular endothelial cells are due to a reduction in endoplasmic reticulum stress [26] and stimulation of vasodilatory and protective nitric oxide (NO) production [27]. In contrast, deletion of the VDR leads to increased transport of cholesterol into the atherosclerotic plaque by monocytes, increased foam cell formation, and an increased number of proatherogenic M2 macrophages within the atherosclerotic plaque [28]. Calcitriol furthermore induces regulatory T cells—which act as anti-atherosclerotic agents [29]—and suppresses neutrophil-induced inflammatory responses [30]. In addition, it reduces the expression of pro-inflammatory cytokines in isolated blood monocytes, which leads to a reduction in acute-phase protein synthesis and may affect the process of atherosclerotic plaque formation [31,32].

The results of our recent study showed significantly elevated values for markers of subclinical inflammation based solely on blood morphotic elements (the Systemic Inflammation Reaction Index, SII; the Systemic Inflammation Response Index, SIRI) in patients with a diagnosis of myocardial infarction compared to patients with chronic coronary syndrome [33]. Furthermore, the group of patients with a diagnosis of acute coronary syndrome was characterized by a negative correlation between the values of both analyzed markers and 25-hydroxyvitamin D (25(OH)D) concentrations [34].

The aim of the present study was to assess the differences in values of a marker depicting inflammation and lipid metabolism (neutrophil/HDL-c ratio) and serum 25(OH)D levels in patients with angiographically confirmed coronary artery disease in relation to the diagnosis of chronic versus acute coronary syndrome (CCS vs. ACS). The values of both parameters analyzed were assessed in patients with different subtypes of ACS: unstable angina (UA), ST-segment elevation myocardial infarction (STEMI), and non-ST-segment elevation myocardial infarction (NSTEMI). In addition, NHR biomarker values and serum 25(OH)D levels were analyzed in subgroups of patients with different degrees of coronary atherosclerosis.

## 2. Materials and Methods

### 2.1. Materials

An analysis was performed with 404 cases of men and 236 women who experienced chest pain and underwent coronary angiography between 2013 and 2017 at Bielański Hospital in Warsaw. The research was accepted by the Bioethical Committee of the Medical University of Warsaw. All these patients gave written consent for their data to be included in this study. Patients with the following were excluded: active cancer; paraneoplastic syndromes; chronic kidney disease in stages G3–G5; immobilized; taking vitamin D supplementation; thyroid disease; active infection of viral or bacterial origin; elevated leukocyte sedimentation rate and/or erythrocyte sedimentation rate, with a blood cell count above 10,000 cells/μL and/or CRP above 5 mg/L. Each patient received a dose of 20 mg rosuvastatin or 40 mg atorvastatin.

### 2.2. Clinical and Laboratory Data

Body mass index (BMI, kg/m^2^) was determined by measurements from an electronic scale and telescopic tape measure (Bielskie waga, Żywiec, Poland). To assess smoking habits, patients were divided into three categories: smokers, former smokers, and non-smokers. Smokers were included in the smokers category if they reported that they smoked daily or less frequently and had smoked more than 100 cigarettes in their lifetime. Former smokers included those who reported not smoking for more than a year and had smoked more than 100 cigarettes in the past. On the contrary, non-smokers were the group who declared that they had never smoked or had smoked less than 100 cigarettes. Pre-diabetes and diabetes were defined as a fasting glucose between 100 and 125 mg/dL or an abnormal result between 140 and 200 mg/dL 2 h after glucose ingestion. A diagnosis of diabetes was made with two consecutive measurements of fasting glucose concentrations above 126 mg/dL or an adiposity glucose above 200 mg/dL and polyuria, polydipsia, polyphagia, or a result above 200 mg/dL after a 2-hour glucose tolerance test. Hyperlipidaemia was found at total cholesterol levels above 200 mg/dL and/or triglyceride levels above 150 mg/dL. In contrast, dyslipidaemia occurs at LDL above 70 mg/dL and HDL below 50 mg/dL. Hypertension was defined as systolic blood pressure above 140 mmHg and/or diastolic blood pressure of 90 mmHg in at least two office measurements. Also, if the mean systolic and diastolic blood pressure values were 135 mmHg and 85 mmHg, respectively, outside the office, hypertension was defined. Blood samples were taken from the elbow vein on an empty stomach within the first 24 h of hospital admission. Total blood cell count (measured until 10 April 2014 with the SYSMEX XT2000i analyzer; from 11 April 2014 SYS-measured with the MEX XN1000 analyzer, SYSMEX, Kobe, Japan), lipid profile (measured with the Cobas Integra 400 Plus, Roche Diagnostics, Rotkreuz, Switzerland), and serum 25(OH)D concentration were measured to calculate the NHR. For patients with triglyceride levels below 400 mg/dL, the Friedewald equation was used to calculate LDL cholesterol. For the rest of the cases, a direct laboratory test was performed. Serum vitamin D concentration was determined using a chemiluminescent immunoassay based on the DiaSorin LIAISON^®^ 25 OH Vitamin D TOTAL Assay. The test results were converted from units of nanograms per milliliter (ng/mL) to nanomoles per liter (nmol/L) according to a factor of 2.5 (1 ng/mL = 2.5 nmol/L). Vitamin D deficiency was divided into severe (less than 10 ng/mL), moderate (10–20 ng/mL), mild (20–30 ng/mL), or no deficiency (more than 30 ng/mL).

Coronary arteriography from radial or femoral access was used to assess the coronary arteries, and partial flow reserve was additionally used in unclear cases. Outcomes were assessed using the Coronary Artery Surgery Study (CASS) scale. Stenosis over 70% in the right coronary artery, circumflex branch, or anterior descending branch—1 point. By comparison, stenosis of more than 50% in the left main coronary artery—2 points. The diagnosis of acute coronary syndrome (ACS) was made with elevated levels of myocardial necrosis markers and, in addition, one of the following symptoms: myocardial ischaemia, recent ischaemia, or new loss of viable myocardium or pathological Q-waves on the ECG. Imaging studies and the presence of a thrombus on angiography were helpful in making the diagnosis.

### 2.3. Statistical Analysis

Shapiro–Wilk tests were performed to assess the normality of the data, and Pearson’s chi-square test or Fisher’s exact test was used to determine differences between groups, depending on the distribution of the data. Continuous variables between the two groups were compared using the Mann–Whitney test. The Kruskal–Wallis test was used to compare results within groups. For variables with a normal distribution, a logarithmic transformation was performed, followed by multiple regression analysis. Multiple regression analysis was employed to identify factors associated with the outcome variable. Model fitness was checked by using the Hosmer–Lemeshow goodness-of-fit test. To express the performance of the logistic regression models, the area under the curve (AUC) statistic was used. The receiver operating characteristic (ROC) curve analysis was used to identify the optimal cut-off values of markers according to Youden’s index (maximum = sensitivity + specificity − 1). A *p*-value < 0.05 was considered statistically significant. Statistical significance was achieved when the two-sided *p*-value was less than 0.05. The software used for the analysis included Statistica 13 (StatSoft Inc., Tulsa, OK, USA) and PQStat 1.8.4 (PQStat Software, Poznan, Poland).

## 3. Results

The Results Section is divided into four sub-sections: (1) information about the study participants; (2) differences in the NHR between patients with different diagnoses and CAD stages; (3) differences in vitamin D concentration data between patients with different diagnoses and CAD stages; and, finally, (4) multivariate regression analysis to identify factors associated with the NHR level.

### 3.1. Characteristics of Participants

A comprehensive description of the patients’ characteristics is presented in Table 1—data are presented as numbers (%) or medians (interquartile ranges).

### 3.2. Differences in NHR and Vitamin D Concentrations Between Patients with Different CAD Stages

There were significant differences between patients with different CASS scores for sex distribution, age, diagnosis status, presence of previous MI, HDL, hypertension, smoking status, NHR, and serum 25(OH)D (Table 2). Patients with CASS 3 were significantly older than patients with CASS 2 (*p* = 0.016). Patients with CASS 0 presented significantly higher HDL, higher serum 25(OH)D, and lower NHR than patients with CASS 1, 2, and 3 (*p* < 0.01 for all). There were no covariates that had a significant influence on the results for the NHR.

### 3.3. Differences in Vitamin D Concentrations Between Patients with Different Diagnoses and CAD Stages

There were significant differences between patients with different diagnoses for sex distribution, age, presence of previous MI, HDL, hypertension, smoking status, neutrophils, NHR, and serum 25(OH)D (Table 3). Table 4 presents the differences in analyzed parameters between patients with serum 25(OH)D value below and above the median value for the population.

### 3.4. Determinants of the NHR Level

Proposed determinants explained the NHR variance in 9% (R2 = 0.09, *p* < 0.001) (Table 5). Sex, BMI, diagnosis, and CASS score turned out to be significant determinants of the NHR level.

The cut-off value, corresponding sensitivity, and specificity for the NHR for the diagnosis of ACS were 0.10, 0.382, and 0.616, respectively. The area under the curve (AUC) for the NHR was 0.63 (95%CI: 0.59–0.68; *p* < 0.001). Table 6 presents differences in the analyzed parameters between patients with an NHR value below and above the median value for the population.

## 4. Discussion

The present study analyzed neutrophil/HDL-C ratio values in groups of cardiac patients with a diagnosis of chronic and acute coronary syndrome. In a cohort of more than 600 patients with chest pain, significantly higher titers of the systemic inflammatory exponent were observed for diagnoses of myocardial infarction (ACS vs. CCS). The threshold NHR for the diagnosis of ACS was set at 0.1. Assessment of infarct types showed the highest NHR value for the diagnosis of ST-segment elevation ACS (STEMI). Although reduced 25(OH)D levels were noted in the entire study group, significantly lower values were presented by patients diagnosed with non-ST-segment elevation and ST-segment elevation myocardial infarction (NSTEMI and STEMI). Analysis of the severity of coronary atherosclerosis using the simple CASS scale showed a lower NHR and higher vitamin D level in patients without significant stenosis compared to the group with tight stenosis of at least one major epicardial artery (CASS 0 vs. CASS 1–3). This is another in a series of papers on the use of markers derived from simple laboratory tests in the diagnosis of CCS and complications. So far, we have observed higher values of inflammatory markers based on parameters derived from blood counts in patients diagnosed with acute syndrome [33,35].

The NHR index depicting inflammatory activity and dyslipidemia, both variables of key importance in the pathogenesis of coronary artery disease, is a relatively new biomarker. The amount of work to date analyzing its association with CAD progression and acute coronary syndrome complications is limited [17,36,37,38,39]. The role of the marker in the diagnosis of ACS is indicated by both the results of our study and the work of other authors highlighting the predictive importance of the NHR in both short- and long-term prognoses. A recent study of a cohort of more than half a thousand patients with ST-segment elevation myocardial infarction treated with primary coronary angioplasty defined the NHR as an independent predictor of in-hospital adverse cardiovascular events (MACE) [17]. There was a significant inverse correlation between the marker value and the risk of cardiac arrest, malignant arrhythmia, cardiac death, stent thrombosis, or myocardial rupture during hospitalization. On the basis of a nearly two-year follow-up of a group of approximately 500 patients with ACS, a minimum index value was established (≥5.74), above which the risk of death and reinfarction in patients over 65 years of age increases significantly. The authors of the cited paper highlighted the greater prognostic value of NHR over monocyte/HDL-C and LDL-C/HDL-C ratios. Similar findings were presented by Ozgeyik et al. [18]. A retrospective study of patients with ACS and complete occlusion of at least one coronary artery demonstrated the superiority of the neutrophil/HDL-C marker over other readily available biochemical parameters in the prediction of MACE in patients after infarct-related artery angioplasty. Both the results of this study and the work of other groups suggest the possibility of using the value of a systemic inflammatory exponent in predicting ACS [38,39]. An analysis by Ren et al. of a cohort of 400 patients with type 2 diabetes identified an NHR titer of ≥4.32, which, with a sensitivity of 65.45% and specificity of 66.19%, correlated with the risk of ACS in this group of patients [39]. Note the differences in the minimum values of the analyzed biomarker suggested by us and other authors as predictors of ACS, which are most likely due to the nature of the patient groups analyzed (ethnicity, comorbidities, etc.). Analogous to our observations, Ren et al. also reported a higher NHR in the subgroup of patients with ST-segment elevation ACS (STE-ACS), highlighting the stronger diagnostic power of the biomarker in the subgroup of STE-ACS patients compared to non-ST-segment elevation ACS (NSTE-ACS) patients (*p* < 0.001). An interesting finding was also provided by a more than 12-year observational study of a group of 130,000 respondents [38], which showed an independent association of elevated NHR or fasting blood glucose levels with a higher risk of adverse cardiovascular events (myocardial infarction, stroke) in patients without diabetes or pre-diabetes. In summary, the results of our work and the studies cited above indicate the applicability of the neutrophil/HDL-C ratio in stratifying the short- and long-term risk of major adverse cardiovascular events.

In the study presented here, we observed a significant positive correlation of the NHR with significant stenosis of at least one epicardial artery (CASS 0 vs. CASS 1–3). Results confirming our observations and indicating a role for the neutrophil/HDL-C ratio in predicting the presence of significant stenosis in the coronary bed have been reported by several authors to date. An analysis by Başyiğit et al. of more than three-hundred patients with ischemia documented by myocardial perfusion testing showed a significant association of higher NHR values with moderate-to-severe coronary artery stenosis [15]. In contrast, Kou et al., in a study of a cohort of more than 400 patients with angiographically confirmed CAD, defined the NHR index as an independent predictor of severe coronary stenosis. The authors used a modified Gensini scale with a stenosis significance criterion of 50% of the arterial lumen to assess the severity of coronary atherosclerosis. Our analysis based on the simple CASS scale considers 70% stenosis of one of the main epicardial arteries or 50% stenosis of the LTW trunk as significant. Nevertheless, irrespective of the criteria adopted, the results of both papers consistently indicate a role for the neutrophil/HDL-C ratio in predicting the presence of significant stenosis of large coronary arteries [12]. Interestingly, Ozgeyik et al. suggest an association of high NHR values with atherosclerotic lesion localization. They found correlations of high NHR values with the proximal location of significant stenosis in patients with myocardial infarction; however, the authors—analogous to our results—did not observe an association with the number of coronary arteries involved, as suggested by another group. In opposition to the results of our study and the work of other authors cited above that indicate a role for the NHR in predicting significant coronary artery stenosis are the results of a study of a relatively small group of patients (64 subjects). The analysis of the 64 subjects showed no correlation of biomarker values with the severity of coronary artery disease on the Gensini scale. Among the inflammatory markers studied, the authors identified IL-25 and MHR as stronger markers for assessing the severity of coronary artery disease; however, in our opinion, the main limitation of the presented results is the size of the study group [14].

In the next stage of our study, the association of serum 25(OH)D levels with ACS diagnosis and the NHR biomarker values was assessed. The reported reduced vitamin D levels in the CAD group as well as the correlation of lower values of the hormone with the presence of significant coronary artery stenosis and myocardial infarction episodes have already been reported by both our team and other investigators. Given the discrepancy between the conclusions of observational studies suggesting a role for calcitriol in the pathogenesis of CAD [40,41,42] and the results of meta-analyses of large intervention trials indicating a lack of benefit of vitamin D supplementation in CVD prevention [43], the topic of the importance of calcitriol in cardiovascular disease is still open. Recently, the need to take into account, in addition to vitamin D levels, the level of klotho protein (a biomarker of its activation and metabolism) when deciding on vitamin D supplementation has been highlighted [44]. Furthermore, in the context of recent reports, it seems crucial to accurately characterize the patient groups likely to benefit from calcitriol treatment. A multi-year follow-up of nearly 20,000 hypertensive patients identified a positive effect of supplementation in CVD prevention only for patients without diabetes and cardiovascular disease [45]. It noted the lack of effect in generally healthy, active, largely vitamin D-rich older people. At the same time, there is growing evidence suggesting an effect of calcitriol on lipid metabolism and inflammatory activity. Analysis of the proteomic profiles of 274 participants from the Qatar Biobank database [46] showed reduced levels of HDL-related apolipoproteins (ApoM and ApoD) in patients with dyslipidemia and concomitant vitamin D deficiency. In addition, this group was characterized by increased expression of acute phase proteins associated with inflammation. Recently, the effect of vitamin D on the function of two human chromosomal loci (Chr17q12-21.1 and Chr17q21.2) associated with chronic inflammatory and autoimmune diseases has been documented [47]. The above-mentioned findings analyzing the mechanisms of the influence of 25(OH)D on HDL-C metabolism and inflammatory activity are reflected in the results of our study. In the present study, the association of the NHR biomarker value as an exponent of subclinical inflammation with 25(OH)D concentration was evaluated for the first time, demonstrating a negative correlation of both variables studied. The possible effect of calcitriol on HDL cholesterol fractions and neutrophil function suggested in our manuscript may explain the correlation observed in the study between low 25(OH)D concentration and higher MHR. However, the possibility of a bidirectional association between these variables—where the MHR value is a predictor of vitamin D concentration—should be considered, which requires further well-designed studies.

In conclusion, both our findings and those of other groups highlight the importance of vitamin D in various areas of health, suggesting its potential impact on low-grade inflammatory activity, mortality risk, lipid metabolism, and inflammation [48]. Research suggests that optimizing vitamin D levels may be beneficial in reducing the risk of death and preventing disease, with potential implications for treatment and prevention strategies. However, further research is needed to better understand the mechanisms underlying these compounds and to determine optimal vitamin D levels for different patient groups.

## 5. Limitations of This Study

The cross-sectional and observational nature of this study made it impossible to assess the causal relationship and analyze the mechanism of influence of the analyzed variables. The sample comprised a limited number of exclusively Polish patients, with no matching of subgroups, and with different diagnoses. The laboratory analysis was based only on cholecalciferol concentration without taking into account calcitriol, ferritin, inflammatory cytokines, HOMA IR index, or glycated hemoglobin concentration. Body composition analysis was also not considered. In addition, coronary artery status was described using the simple CASS scale, without taking into account the amount of calcification or the effect of hypolipemic treatment (type of statin or duration of treatment).

## 6. Conclusions

Despite decades of intensive research, we are still looking for new risk factors and treatments for chronic and acute coronary syndromes. Given the key role of systemic inflammation in the process of atherosclerosis and its complications, new, readily available inflammatory markers could help identify patients at increased risk of CVD. The demonstrated association of NHR with the diagnosis of significant coronary artery stenosis and myocardial infarction may suggest the possibility of using the biomarker as an additional criterion in the diagnosis of patients with chest pain. The observed significant differences in its values in the categories of chronic patients and acute coronary syndromes suggest the consideration of immunosuppressive drugs with a systemic mechanism of action as a major tool in the treatment of CAD.

We also note the possibility of using a biomarker in assessing treatment efficacy, which requires further extensive research. At the same time, in spite of the commitment of millions of researchers, we still do not know the definitive definition of vitamin D deficiency or sufficiency. Moreover, we are uncertain about the cardiovascular consequences of deficiency and the benefits of improving the body’s calcitriol stores. However, considering the well-documented effect of vitamin D on inflammatory processes, the negative correlation we have shown with the NHR, and the significantly lower levels accompanying the diagnosis of ACS, we consider a potential bidirectional relationship of the analyzed variables, which should be urgently clarified.

## Figures and Tables

**Table 1:** Characteristics of participants.

Variable	Values
N of participants [♂/♀]	404 (63.1%)/236 (36.9%)
Age [years]	66.4 (59.3–74.9)
BMI [kg/m^2^]	27.7 (24.9–31.1)
Diagnosis [stable IHD/STEMI/NSTEMI/UA]	335 (52.3%)/143 (22.3%)/97 (15.2%)/65 (10.2%)
Previous MI [yes/no]	249 (38.9%)/391 (61.1%)
High-density lipoprotein (HDL) (mg/dL)	47.1 (39.2–57.7)
Hypertension [yes/no]	534 (83.4%)/106 (16.6%)
Smoking [active/former smoker/no]	182 (28.4%)/71 (11.1%)/353 (55.2%)
Type 2 diabetes mellitus [yes/pre-diabetes/no]	218 (34.1%)/26 (4.1%)/394 (61.6%)
Coronary Artery Surgery Study (CASS) scale [0/1/2/3]	158 (24.7%)/176 (27.5%)/161 (25.2%)/145 (22.7%)
Neutrophils [thousand cells/µL]	4.8 (3.7–6.2)
NHR	0.10 (0.07–0.14)
Serum 25(OH)D (ng/mL)	15.1 (10.2–21.2)

**Table 2:** Association between parameters and CAD stages.

Variable	CASS 0	CASS 1	CASS 2	CASS 3	*p*
N of participants [♂/♀]	72/86	115/61	118/43	99/46	<0.001
Age [years]	67.0 (59.7–72.4)	65.2 (58.6–75.3)	63.6 (58.4–74.8)	69.2 (62.0–76.4)	0.015
BMI [kg/m^2^]	27.7 (24.8–31.6)	27.2 (24.8–31.0)	27.7 (24.9–30.5)	28.1 (25.3–30.8)	0.815
Diagnosis [stable IHD/STEMI/NSTEMI/UA]	133/7/10/8	64/63/31/18	76/38/29/18	62/35/27/21	<0.001
Previous MI [yes/no]	12/146	73/103	79/82	85/60	<0.001
High-density lipoprotein (HDL) (mg/dL)	53.8 (43.2–64.1)	46.6 (39.4–55.5)	46.7 (38.4–55.1)	44.3 (36.5–52.7)	<0.001
Hypertension [yes/no]	118/40	144/32	144/17	128/17	0.001
Smoking [active/former smoker/no]	27/11/109	62/17/87	56/22/78	37/21/79	<0.001
Type 2 diabetes mellitus [yes/pre-diabetes/no]	47/7/103	51/4/120	59/9/93	61/6/78	0.089
Neutrophils [thousand cells/µL]	4.5 (3.6–5.7)	4.8 (3.8–6.4)	4.7 (3.7–6.2)	5.0 (3.9–6.5)	0.107
NHR	0.09 (0.06–0.12)	0.11 (0.08–0.15)	0.11 (0.08–0.15)	0.11 (0.08–0.17)	<0.001
Serum 25(OH)D (ng/mL)	17.7 (11.6–24.2)	14.2 (9.8–19.8)	13.9 (11.0–18.3)	14.6 (9.3–20.8)	<0.001

**Table 3:** Association between parameters and diagnosis.

Variable	Stable IHD	STEMI	NSTEMI	UA	*p*
N of participants [♂/♀]	206/129	107/36	59/38	32/33	0.002
Age [years]	67.3 (60.5–75.5)	62.4 (56.2–69.2)	65.6 (58.2–75.9)	70.4 (62.9–76.4)	<0.001
BMI [kg/m^2^]	27.9 (24.9–31.1)	26.8 (24.7–31.0)	27.7 (24.8–31.7)	27.9 (24.8–30.4)	0.502
Previous MI [yes/no]	102/233	69/74	57/40	21/44	<0.001
High-density lipoprotein (HDL) (mg/dL)	50.2 (40.8–61.4)	45.0 (36.6–52.3)	42.6 (35.3–50.6)	46.5 (39.6–55.4)	<0.001
Hypertension [yes/no]	270/65	117/26	84/13	63/2	0.009
Smoking [active/former smoker/no]	66/53/196	67/8/63	39/3/48	10/7/46	<0.001
Type 2 diabetes mellitus [yes/pre-diabetes/no]	120/15/199	42/3/97	31/6/60	25/2/38	0.454
Neutrophils [thousand cells/µL]	4.7 (3.6–5.8)	5.3 (4.1–7.2)	5.0 (3.8–6.1)	4.8 (3.7–6.5)	0.003
NHR	0.09 (0.07–0.13)	0.12 (0.09–0.17)	0.11 (0.09–0.16)	0.11 (0.08–0.13)	<0.001
Serum 25(OH)D (ng/mL)	16.2 (11.1–22.2)	13.9 (9.9–18.1)	13.4 (9.6–19.3)	17.5 (11.1–22.0)	0.007

**Table 4:** Differences in analyzed parameters between patients with serum 25(OH)D values below and above the median value for the population.

Variable	Serum 25(OH)D < 15.1	Serum 25(OH)D ≥ 15.1	*p*
N of participants [♂/♀]	317 [187/130]	323 [217/106]	0.032
Age [years]	65.5 (58.1–74.9)	66.9 (60.2–75.3)	0.132
BMI [kg/m^2^]	27.7 (24.8–31.3)	27.7 (25.1–30.5)	0.967
Diagnosis [stable IHD/STEMI/NSTEMI/UA]	143/89/55/30	192/54/42/35	<0.001
Previous MI [yes/no]	133/184	116/207	0.117
High-density lipoprotein (HDL) (mg/dL)	45.9 (38.2–55.6)	48.7 (39.9–58.9)	0.036
Hypertension [yes/no]	270/47	264/59	0.242
Smoking [active/former smoker/no]	109/24/184	73/47/203	<0.001
Type 2 diabetes mellitus [yes/pre-diabetes/no]	114/17/186	104/9/210	0.115
Coronary Artery Surgery Study (CASS) scale [0/1/2/3]	57/94/90/76	101/82/71/69	0.001
Neutrophils [thousand cells/µL]	4.9 (3.9–6.3)	4.7 (3.5–6.2)	0.016
NHR	0.11 (0.08–0.16)	0.09 (0.07–0.13)	0.002

**Table 5:** Determinants of NHR level.

Determinants	β (±95% CI)	*P*
Age [years]	−0.06 (−0.14–0.03)	0.182
Sex	0.13 (0.05–0.21)	0.002
BMI [kg/m^2^]	0.12 (0.04–0.20)	0.004
Diagnosis	0.13 (0.05–0.22)	0.001
CASS score	0.12 (0.03–0.21)	0.009
Previous MI [yes/no]	0.06 (−0.02–0.14)	0.160
Hypertension [yes/no]	−0.07 (−0.15–0.01)	0.087
Smoking [active/former smoker/no]	0.02 (−0.06–0.11)	0.582
Type 2 diabetes mellitus [yes/pre-diabetes/no]	0.06 (−0.02–0.14)	0.125

**Table 6:** Differences in analyzed parameters between patients with NHR value below and above the median value for the population.

Variable	NHR < 0.1	NHR ≥ 0.1	*p*
N of participants [♂/♀]	312 [176/136]	328 [228/100]	<0.001
Age [years]	67.3 (60.6–75.4)	64.5 (57.7–74.8)	0.010
BMI [kg/m^2^]	27.5 (24.2–30.8)	27.8 (25.5–31.2)	0.079
Diagnosis [stable IHD/STEMI/NSTEMI/UA]	198/47/37/30	137/96/60/35	<0.001
Previous MI [yes/no]	104/208	145/183	0.005
High-density lipoprotein (HDL) (mg/dL)	54.8 (46.3–64.1)	40.7 (34.7–48.4)	<0.001
Hypertension [yes/no]	261/51	273/55	0.886
Smoking [active/former smoker/no]	68/42/202	114/29/185	<0.001
Type 2 diabetes mellitus [yes/pre-diabetes/no]	96/13/203	122/13/193	0.228
Coronary Artery Surgery Study (CASS) scale [0/1/2/3]	101/78/76/57	57/98/85/88	<0.001
Neutrophils [thousand cells/µL]	3.8 (3.2–4.6)	5.9 (4.9–7.5)	<0.001
Serum 25(OH)D (ng/mL)	16.5 (11.1–22.1)	14.0 (9.9–20.0)	0.023

## Data Availability

Data can be provided by the corresponding author upon reasonable request.
